# Validation experiments on finite element models of an ostrich (*Struthio camelus*) cranium

**DOI:** 10.7717/peerj.1294

**Published:** 2015-10-13

**Authors:** Andrew R. Cuff, Jen A. Bright, Emily J. Rayfield

**Affiliations:** 1 GEE, University College London, London, United Kingdom; 2 Structure and Motion Laboratory, The Royal Veterinary College, Hatfield, United Kingdom; 3 School of Earth Sciences, University of Bristol, Bristol, United Kingdom; 4 Department of Animal and Plant Sciences, University of Sheffield, Sheffield, United Kingdom

**Keywords:** Ostrich, Skull, Validation, Strain, Finite element analysis

## Abstract

The first finite element (FE) validation of a complete avian cranium was performed on an extant palaeognath, the ostrich (*Struthio camelus*). *Ex-vivo* strains were collected from the cranial bone and rhamphotheca. These experimental strains were then compared to convergence tested, specimen-specific finite element (FE) models. The FE models contained segmented cortical and trabecular bone, sutures and the keratinous rhamphotheca as identified from micro-CT scan data. Each of these individual materials was assigned isotropic material properties either from the literature or from nanoindentation, and the FE models compared to the *ex-vivo* results. The FE models generally replicate the location of peak strains and reflect the correct mode of deformation in the rostral region. The models are too stiff in regions of experimentally recorded high strain and too elastic in regions of low experimentally recorded low strain. The mode of deformation in the low strain neurocranial region is not replicated by the FE models, and although the models replicate strain orientations to within 10° in some regions, in most regions the correlation is not strong. Cranial sutures, as has previously been found in other taxa, are important for modifying both strain magnitude and strain patterns across the entire skull, but especially between opposing the sutural junctions. Experimentally, we find that the strains on the surface of the rhamphotheca are much lower than those found on nearby bone. The FE models produce much higher principal strains despite similar strain ratios across the entirety of the rhamphotheca. This study emphasises the importance of attempting to validate FE models, modelling sutures and rhamphothecae in birds, and shows that whilst location of peak strain and patterns of deformation can be modelled, replicating experimental data in digital models of avian crania remains problematic.

## Introduction

The finite element (FE) method is now widely used in biology and palaeontology as a tool for ascertaining stress, strains, and deformation in biological structures. Commonly used in engineering, the method involves defining a complex object as a large number of geometrically simple shapes (elements). These elements are connected by nodes at their corners (and sometimes midpoints) in a finite element mesh (Zienkiewicz, Taylor & Zhu, 2005). Accurate FE models can provide an insight into biomechanical performance, and potentially help define the link between form and function, a long standing debate in comparative biology and palaeontology. The output of the model is dependent on the input parameters, such as material properties, geometry, and loading; inaccuracies in these input parameters will be compounded in the output, and as such may give results that are a poor reflection of how the structure behaves in reality. Material properties in biological structures are highly complex. Cranial bone, for example, has heterogeneous mechanical properties and is anisotropic depending on the location being examined (Peterson & Dechow, 2003; Wang & Dechow, 2006; Dechow, Wang & Peterson, 2010; Zapata et al., 2010; Soons et al., 2012a).

One way to test the reliability of FE models is to compare their results to those garnered from *in vivo* or *ex vivo* validation experiments. As the majority of non-clinical FE studies to date have focussed on crania, the majority of non-clinical validation studies have also addressed this structure. The cranium is highly complex, and is made up of many materials (e.g., cortical bone, cancellous bone, sutures, teeth), the influence of which has been tested in FE models of some taxa (Strait et al., 2005; Kupczik et al., 2007; Davis et al., 2011). The majority of cranial validation studies have been carried out in primates, but other studies have included pigs (Bright & Rayfield, 2011a) and alligators (Metzger, Daniel & Ross, 2005; Porro et al., 2013). Like the majority of modern and extinct vertebrates, these animals all have teeth. Only three validation studies have been performed on edentulous animals, an ostrich lower jaw (which treated the mandible as a single material, bone, and did not include the rhamphotheca; Rayfield, 2011), and two studies on finch beaks (Soons et al., 2012b; Soons et al., 2012c).

There is still much to understand about modelling bird crania. Bird skulls are particularly complex as they possess very thin cortical bone, diffuse trabeculae and open (patent) sutures, all of which have no published material properties. In addition, birds possess a flexible keratinous rhamphotheca, found in all extant birds, as well as extant and extinct turtles, platypuses, echidnas, and are believed to occur in many extinct archosaurs (all major dinosaurian clades, pterosaurs, crocodilians, crurotarsans) and dicynodonts (Louchart & Viriot, 2011). Keratin possesses a lower Young’s modulus than bone and as such is predicted to affect biomechanical performance (Soons et al., 2012a; Soons et al., 2012b; Soons et al., 2012c; Lautenschlager et al., 2013)

The effect of cranial sutures on skull performance must also be considered. Sutures exist as a soft tissue connection between the bones in all cranial elements of younger animals, but some sutures are lost as the bones fuse through ontogeny. However, not all sutures fully fuse and as such potentially introduce weak spots in the cranium. Sutures may have a biomechanical function, for example modulating strains, and/or acting as strain sinks (Jaslow, 1990; Herring & Mucci, 1991; Jaslow & Biewener, 1995; Moazen et al., 2009; Moazen, Costantini & Bruner, 2013; Curtis et al., 2013).

This study performs a validation experiment on an FE model of the cranium of an ostrich (*Struthio camelus*), a large, extant, herbivorous avian palaeognath native to Africa. We hypothesise that our FE-model, of the same experimental specimen and with controlled boundary conditions, can reproduce experimentally recorded strain values. In particular we test the influence of material property choice, sutures, and the introduction of a keratinous rhamphotheca on the FE model outputs. We also predict that introducing more sophisticated properties and geometry to our FE models will increase the model accuracy with reference to the replication of measured *ex-vivo* strains.

## Materials and Methods

### *Ex-vivo* experimentation

An ostrich skull, frozen with jaws in occlusion (a farmed sub-adult, aged 6-9 months, obtained after slaughter for meat production), was thawed and the jaw adductor muscles were dissected out (for an overview of avian cranial musculature see Holliday & Witmer, 2007). Ostriches were used due to their large size and the ability to adhere gauges to the surface of the bone, and as they retain patent sutures in some skull regions into adulthood. The M. pseudotemporalis superficialis was isolated, as this muscle provided the largest and most robust attachment site for loading in the experiments. The muscle was then weighed, before immersion in 15% nitric acid, then bisected to measure individual fibre lengths and pennation angle to determine maximum muscle force (after Biewener & Full, 1992).

**Figure 1:**
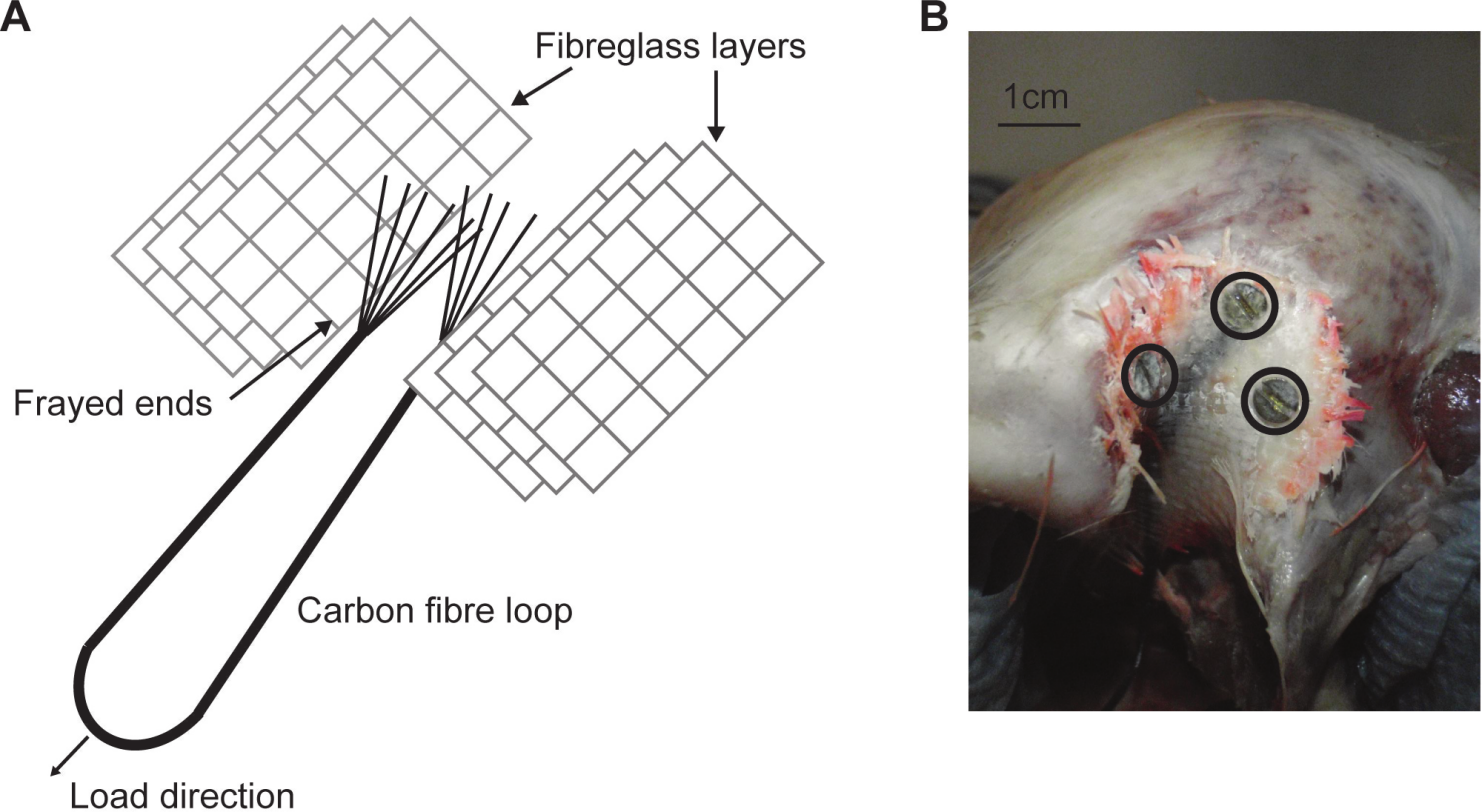
Artificial tendon. (A) Schematic of the artificial tendon construction showing the carbon fibre loop sandwiched between layers of fibreglass. (B) The artificial tendon screwed into place on the M. pseudotemporalis superficialis. Screws highlighted in black circles.

Due to difficulties in loading an *ex-vivo* skull with accurate muscle loads and orientations, a number of methods have been used. These include directly loading the original (albeit formaldehyde preserved) muscles (Kupczik et al., 2007), or screwing material into the skull at the site of muscle attachment on which to apply the forces (e.g., Bright & Rayfield, 2011a). This paper attempts a new method to circumvent some issues associated with previous attempts. Two artificial tendons were made using a strand of carbon fibre, resin and fibreglass. The carbon fibre was looped, with the two loose ends being frayed before being sandwiched between two sets of three fibreglass layers (Fig. 1A). The fibreglass layers were all then impregnated with epoxy resin creating artificial ‘tendons’ that were strong, light, and flexible.

The ‘tendons’ were attached to the M. pseudotemporalis superficialis attachment site (delineated by a bony ridge) on the dorsolateral side of the cranium with cyanoacrylate (M-Bond 200; Vishay Micro-Measurements, Basingstoke, UK), before being screwed in place using three 3.5 mm self-tapping screws (Fig. 1B). Therefore, it should be noted that the loadings replicate only one muscle load and are not truly physiological or representative of a bite. This was considered acceptable, given that the aims of the experiment were specimen-specific validation and testing model sensitivity to the effects of the material properties, sutures and rhamphotheca. During dissection, preparation and testing, the skull and rhamphotheca were kept as moist as possible by frequently covering both with a 50:50 mixture of glycerol and water.

**Figure 2:**
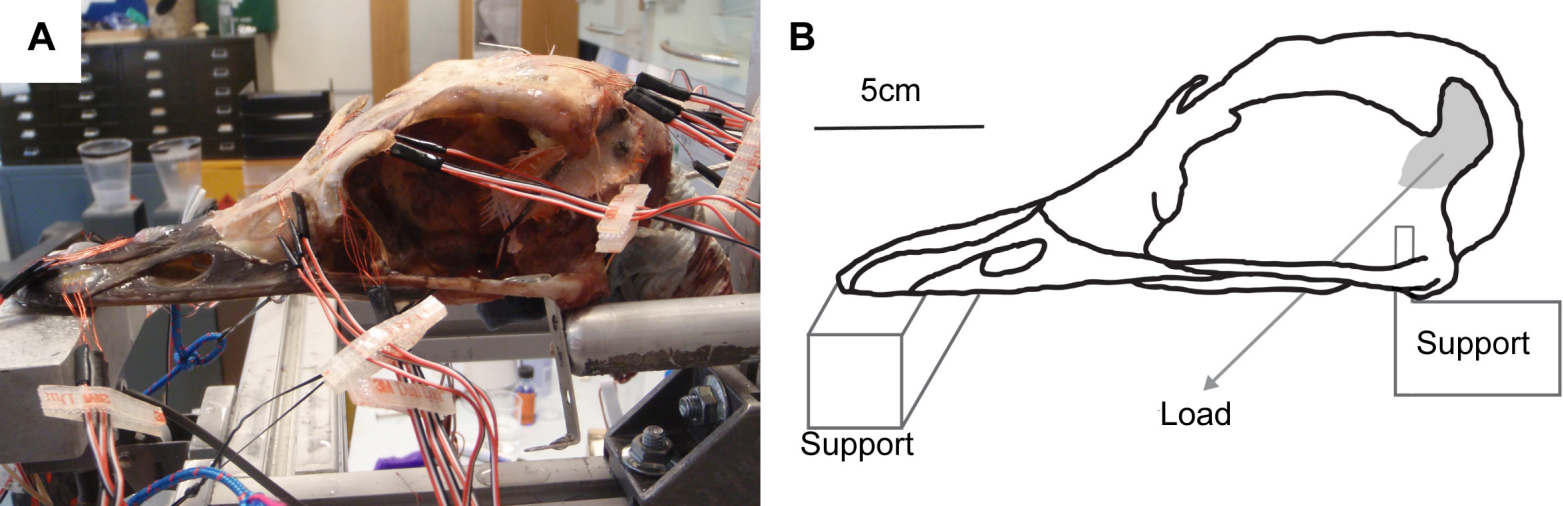
*Ex-vivo* experimental set up. (A) Experimental testing of ostrich with gauges attached, under loading of the artificial tendons. (B) Schematic of experimental rig showing load and constraints.

**Figure 3:**
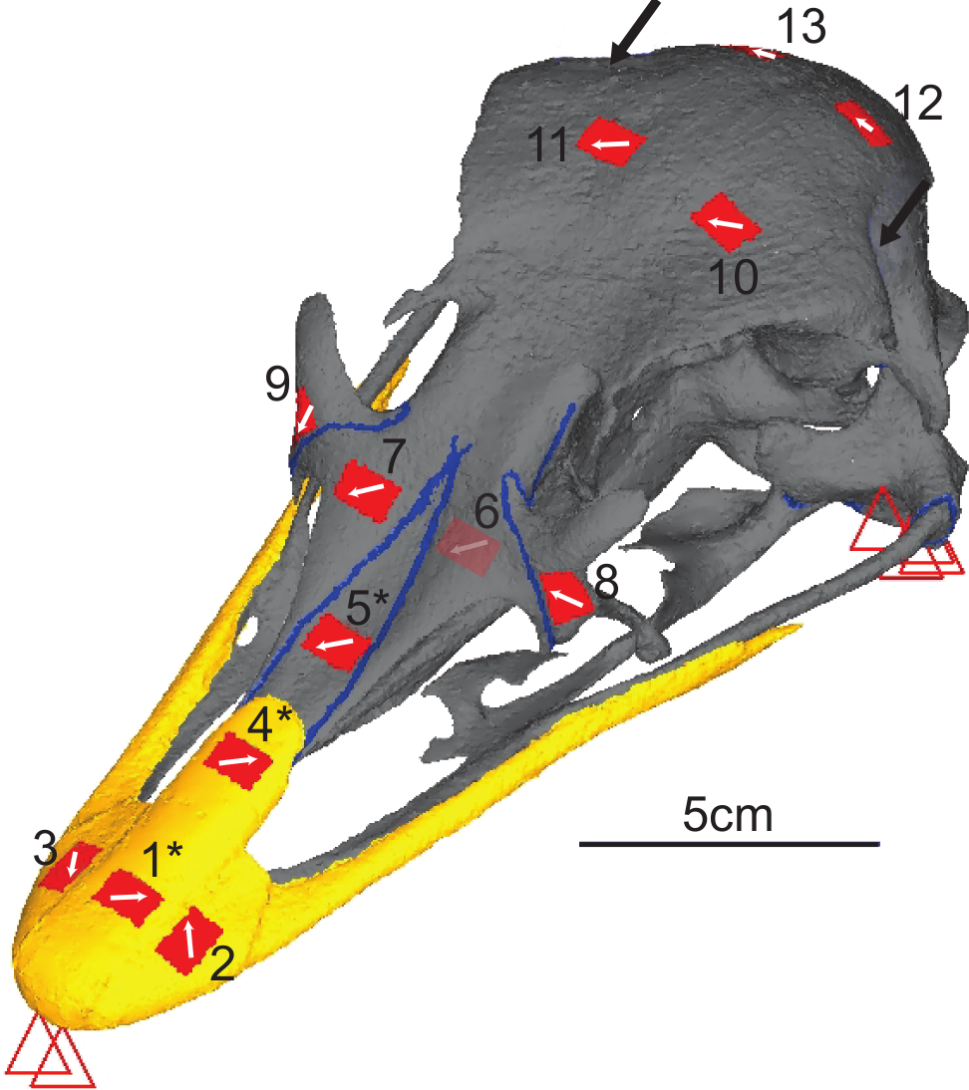
Digital reconstruction of the ostrich skull. Red triangles represent the constraints, black arrows show orientation and location of loads, red rectangles are membrane elements that mirror the strain gauges. Gauge 6 was non-functional so was not included in the model, but its location is marked. The blue lines are sutures, and the yellow material is the keratinous rhamphotheca. The trabecular bone is not visible. Gauges labelled with an asterisk (*) are sites where nanoindentation was performed. Direction from grid one is labelled as the white arrow from which strain orientation were measured.

Rectangular planar rosette strain gauges (C2A-06-062LR-350; gauge length 1.52 mm, backing 7.04 × 10.41 mm, Vishay Micro-Measurements, Basingstoke, UK) were attached at 13 sites across the skull (Figs. 2 and 3). The gauge sites were prepared by removing any remaining periosteum with pumice powder (GC-5 Pumice powder; Vishay Micro-measurements, Basingstoke, UK) and the site cleaned with 95% ethanol. Gauges were attached to the sites with cyanoacrylate adhesive (M-Bond 200; Vishay Micro-Measurements, Basingstoke, UK). The gauges were then covered with a waterproof silicon rubber coating for protection (3140 RTV Coating; Dow Corning, Midland, MI, USA). Strains from the 13 gauges were connected to an amplifier (5100B; Vishay Micro-Measurements, Basingstoke, UK), recorded, and converted to principal strains and strain orientations using STRAINSMART 4.01 software (Vishay Measurements Group, Basingstoke, UK). Preliminary drift tests were conducted for all gauges before loading and no thermal drift was observed over a 30 s time period.

The cranium was placed upon a specially designed loading rig adapted from a previous study (Bright & Rayfield, 2011a) which supported the cranium bilaterally under the quadrate at the quadrate/articular (QA) joint with two aluminium bars, and at the anteriormost part of the rhamphotheca with a flat block (Fig. 2). Each of the aluminium bars rested between the quadrate articular surfaces and prevented dorsoventral and mediolateral movement of the posterior of the cranium under loading. To prevent anterior motion during loading, a small, flat, thin plate was attached to the end of each bar that contacted the anterior ventral quadrate during the test. Loads were applied by tying 3 mm low-stretch polyester cord (3 mm Magic Speed; LIROS GmbH, Berg, Germany) to the carbon fibre loops using bowline knots at one end, and hanging balances (HCB 50K100; Kern & Sohn GmbH, Balingen, Germany) at the other. The cord was extended over low-friction pulleys (Size 1 upright block, Barton Marine Equipment, Kent, UK) to apply the load approximating the muscle line of action. The hanging balances (precision of 1 N) were attached to rigging screws (6 mm Fork Bottlescrew; Sea Sure, Hampshire, UK) which allowed for manual tightening of the screws to apply tension without twisting.

Due to the limitations of the amplifier, only four gauges could be tested at any time. During the testing, channel 3 on gauge 6 failed, so gauge 6 was discounted from the study, thus recordings were taken from three groups of four gauges (G1-G4, G5-G9, G10-G13). Each gauge was zeroed, and loads of 32 N were applied to each side by tightening the rigging screws simultaneously. These forces were within the limits of an estimated force for the M. pseudotemporalis superficialis calculated from the dissection (muscle body = 4.18 g, average fibre length = 18.4 mm, average pennation angle = 30.2°; using a muscle stress of 0.3 Nmm^−2^ gave an average force of 44.7 N for each side) and caused cranial deformation, but not so large that fracture may occur under loading. All gauges were measured twice during two complete trials. After each batch of three or four gauges was loaded, the residual load remaining in the hanging balance after unloading was measured: right tendon average = 6.86 N [S.D. = 1.46], left tendon average = 5.71 N [S.D. = 0.488]. These residual loads probably resulted from friction, knot tightening and elasticity in the cord, and may have resulted in slight variations in the actual loading conditions between each trial. Thus, average residual loads were deducted from the initial load, to give average applied loads of 25.1 N on the right tendon, and 26.3 N on the left tendon. The data from each gauge during a trial is an average of 10 recordings per second taken for 60 s over which a static load was applied (a total of 600 readings per gauge).

### FE models

Prior to dissection, the skull was subject to micro-computed tomography (micro-CT) at the University of Hull, UK using a X-Tek HMX 160 scanner. Due to the size of the specimen, it was scanned in two parts (anterior and posterior skull) taken at slightly different orientations (756 slices and 846 slices, with voxel sizes of 0.1594 mm and 0.1425 mm respectively). Both scan sets were rotated and resampled to the same voxel size (0.1594 mm resolution) in Avizo 6.3 (Mercury Computer Systems, Arlington, Virginia, USA) where the cortical bone, trabecular bone, sutures and rhamphotheca were initially segmented automatically before final details were segmented manually. A resulting stereolithographic surface was then imported into Hypermesh 10.1 (Altair Engineering, Menlo Park, California, USA). Surfaces for each individual material (cortical bone, trabecular bone, suture and rhamphotheca) were meshed using the ‘shrink wrap’ function in Hypermesh. The result was a continuous mesh with the rhamphotheca directly attached to the cortical bone, with the sutures connected to cortical bone (and directly to the rhamphotheca in the places where the rhamphotheca overlies the suture), and many separate enclosed surfaces of trabecular bone located throughout the skull. A surface-based meshing approach was then used to generate 3D volumetric meshes of the cranium. Five meshes of between 1,333,804 and 1,749,533 elements (between 298,515 and 385,811 nodes) were produced for convergence testing (Bright & Rayfield, 2011b) for both Tet4 (four nodes per tetrahedral element) and Tet10 (10 nodes per element) models. The ‘shrink wrap’ function in Hypermesh was unable to produce usable meshes for element numbers outside of these limits for the ostrich cranium. When results at equivalent gauges sites for successive meshes were less than 10% different in maximum and minimum strain, and the difference in total strain energy was less than 5%, the mesh was considered converged. On this basis, the Tet10 mesh with 1,749,533 elements was selected for further analysis (see Supplemental Information for further information). Each model surface was cleaned by removing t-connections, holes, and free edges. Virtual gauges matching the location of the *ex-vivo* gauges were created using 2D membrane elements placed as close to the actual gauge locations as possible, in order to account for any discrepancy between the 3D strains of the models being projected in to the 2D plane of the gauge (Bright & Rayfield, 2011a). Load and constraint points equivalent to those experienced in the *ex-vivo* cranium were applied to the model. Two constraints prevented dorsoventral translation at the anterior of the beak, and four on each quadrate (at the jaw joint) preventing movement in all directions. The model was loaded with a force of 25 N on the right side and 26 N on the left in the orientation specified by the experimentally applied load (Figs. 2 and 3). These loads were distributed across 14 nodes at each muscle attachment site (four nodes at each of the three screw locations to replicate higher point loads induced by the introduction of a screw and two further nodes equidistant between the screw locations).

## Material Properties

For the convergence test, a single isotropic material property was assigned to the entire model (E = 7 GPa, v = 0.35 after Rayfield, 2011). For the subsequent analysis, three types of material property datasets were used: values from the literature; posthoc values selected to attempt to obtain the best fit between peak experimental values and FE models; and values obtained from nanoindentation on an ostrich cranium. Due to a limited availability of avian cranial material properties, a combination of published materials from both birds and mammals were used to assess the influence of using literature values, in some cases from other taxa, on the FE results. These included bird keratin, mammalian sutures, avian femoral cortical bone, and mammalian trabecular bone (Table 1, see footnotes for references). In the posthoc models, the Young’s modulus of each material was sequentially modified until experimental peak strains were matched or exceeded in the FE model.

**Table 1:** Material properties for the FE models. All units for Young’s modulus (*E*) are MPa.

	Cortical bone		Trabecular bone		Suture		Beak	
	*E*	*ν*	*E*	*ν*	*E*	*ν*	*E*	*ν*
**Literature values**								
L1	7,000^a^	0.35^a^	7,000^a^	0.35^a^	7,000^a^	0.35^a^	7,000^a^	0.35^a^
L2	13,620^b^	0.35	0.64^c^	0.28^c^	50^f^	0.40^h^	1,000^i^	0.35
L3	13,620^b^	0.35	0.64^c^	0.28^c^	50^f^	0.40^h^	3,100^j^	0.35
L4	13,620^b^	0.35	1,000^d^	0.30^e^	50^f^	0.40^h^	1,000^i^	0.35
L5	13,620^b^	0.35	1,000^d^	0.30^e^	50^f^	0.40^h^	3,100^j^	0.35
L6	13,620^b^	0.35	2,000^d^	0.30^e^	50^f^	0.40^h^	3,100^j^	0.35
L7	13,620^b^	0.35	1,000^d^	0.30^e^	46^g^	0.35^a^	1,330^k^	0.35
L8	13,620^b^	0.35	2,000^d^	0.30^e^	46^g^	0.35^a^	1,330^k^	0.35
L9	13,620^b^	0.35	2,000^d^	0.30^e^	13,620^b^	0.35^a^	3,100^j^	0.35
**Posthoc values**								
PH1	10,000	0.35	100	0.28	50	0.40	3,000	0.35
PH2	10,000	0.35	1,000	0.28	50	0.40	3,000	0.35
PH3	7,000	0.35	1,000	0.28	50	0.40	3,000	0.35
PH4	7,000	0.35	1,000	0.28	1,000	0.40	3,000	0.35
PH5	7,000	0.35	100	0.28	50	0.40	1,000	0.35
PH6	7,000	0.35	100	0.28	10	0.40	1,000	0.35
PH7	7,000	0.35	50	0.28	10	0.40	1,000	0.35
PH8	5,000	0.35	50	0.28	10	0.40	1,000	0.35
**Nanoindentation values**								
Mean	5,030	0.35	1,500^d^	0.30	27.1	0.35	18	0.35
Max	9,890	0.35	1,500^d^	0.30	67	0.35	46	0.35
Min	1,240	0.35	1,500^d^	0.30	9	0.35	9	0.35

**Notes.**

a Rayfield, 2011—best match to ostrich mandible FE models.

b Yamada, 1970—ostrich femoral bone.

c Ashman, Rho & Turner, 1989—mammalian trabecular bone.

d O’Mahony et al., 2000—human edentulous mandible.

e Teo et al., 2006—porcine vertebral cancellous bone.

f Odame, Yu & Zhang, 2005—mammal suture.

g Rafferty, Herring & Marshall, 2003—pig nasofrontal suture.

h Mow & Huiskes, 2005—Bovine cartilage, Athanasiou et al., 1998—human articular cartilage.

i Seki, Bodde & Meyers, 2010—lowest of values for toucan beak 1.04 ± 0.06 GPa and 1.12 ± 0.13 GPa.

j Soons et al., 2012a—dry finch beak keratin.

k Bonser, 2000—ostrich claw keratin along mediolateral axis.

Nanoindentation studies were performed on samples from a different subadult ostrich cranium (an individual also aged 6–9 months, with the fleshed skull being frozen shortly after slaughter) obtained from the same breeder. Samples were harvested from the cranium in areas approximately 2 cm^2^ in locations equivalent to gauges 1, 4 and 5, using an oscillating saw (Fig. 3). Locations were chosen to give multiple sites for rhamphotheca and bone (to check for variability in properties across the cranium), and to provide suture material property data. Young’s moduli were measured using a nano-hardness tester with a Berkovitch diamond indenter (CSM Instruments S.A., Peseux, Switzerland) at the University of Hull. Mean, maximum, and minimum values obtained from nanoindentation were used in the FE-models to provide the broadest possible range of property variation.

Each model was then analysed in Abaqus 6.8.2 (Dassault Systèmes Stimulia, Providence, RI, USA) on a desktop PC (Windows 64-bit, XP Professional, Intel Xeon x5450 2.00 GHz CPU, 16 GB RAM).

The results of the models were compared to the *ex-vivo* experimental values graphically and by using Euclidean distances (ED), which have been used in previous studies (Kupczik et al., 2007; Kupczik et al., 2009; Bright, 2012). This metric works by showing how similar (or dissimilar) datasets are to one another; the smaller the ED value, the higher the level of similarity between the datasets. In addition to ED values, regression tests were performed. This method tests for significant relationships between the metrics in the various trials (See Supplemental Information for further information).

## Results

### Nanoindentation data

Nanoindentation values for ostrich cranial bone, rhamphotheca and suture are listed in Table 2.

**Table 2:** Young’s modulus (*E*) of various materials on an ostrich skull obtained by nanoindentaion (see text and Fig. 3 for test sites and experimental detail).

	Bone	Beak	Suture
Gauge sites	1,4,5	1,4	5
Indents	59	40	20
Mean (GPa)	5.03	0.0271	0.018
Max (GPa)	9.89	0.067	0.046
Min (GPa)	1.24	0.009	0.009
St Dev (GPa)	1.95	0.0144	0.011
Median (GPa)	4.95	0.0245	0.013

### *Ex-vivo* experimental strain

The maximum (*E_max_*; Table 3), minimum (*E_min_*; Table 4) principal strains, strain ratios (Table 5) and strain orientations (Table 6) show similar trends between the two *ex-vivo* tests, but there is variation in magnitude between tests. The data, however, are significantly correlated for all metrics (see Figs. S3–S9).

**Table 3:** Maximum principal strains for the *ex-vivo* experiment and finite element models. All values are in microstrain.

Gauge site	1	2	3	4	5	7	8	9	10	11	12	13
***Ex-vivo***												
Test 1	17	−22	−8.0	−6.0	−7.0	990	−20	25	4.0	4.0	58	21
Test 2	14	−39	−25	1.0	−77	970	13	14	6.0	6.0	71	31
**Literature**												
L1	74	44	37	140	260	130	0.53	1.4	16	18	5.3	5.4
L2	200	87	81	250	180	190	2.6	3.5	4.6	10	21	22
L3	130	70	67	200	180	180	2.6	3.5	4.5	10	21	22
L4	170	74	66	240	180	180	1.7	2.4	22	25	18	17
L5	120	60	54	190	180	170	1.7	2.4	22	25	18	16
L6	110	58	52	180	180	170	1.5	2.2	19	21	13	12
L7	160	74	66	230	180	180	1.6	2.3	22	25	18	16
L8	150	71	64	230	180	180	1.5	2.1	19	21	13	12
L9	100	52	45	140	140	88	0.56	1.2	18	21	12	11
**Posthoc**												
PH1	150	77	90	230	250	230	2.80	3.9	37	43	39	35
PH2	130	73	84	230	250	230	2.2	3.2	28	31	21	19
PH3	160	87	77	270	360	300	3.0	4.4	36	41	24	23
PH4	150	81	70	240	380	210	1.6	3.7	36	40	24	22
PH5	280	130	110	400	380	340	3.9	5.5	52	60	53	48
PH6	300	150	140	440	300	360	3.1	4.5	52	62	54	49
PH7	300	160	140	440	300	360	3.30	4.67	54	66	58	53
PH8	360	190	170	550	450	490	4.9	6.9	74	87	78	71
**Nanoindentation**												
N1	570	140	160	680	480	470	3.7	4.9	41	44	21	21
N2	310	77	80	350	250	240	2.2	2.9	26	28	17	16
N3	1,600	500	540	2,500	2,100	1,500	9.6	12	84	85	26	27

**Table 4:** Minimum principal strains for the *ex-vivo* experiment and finite element models. All values are in microstrain.

*Gauge site*	1	2	3	4	5	7	8	9	10	11	12	13
***Ex-vivo***												
Test 1	−59	−69	−68	−78	−970	170	−110	−100	−83	−83	−100	−77
Test 2	−85	−120	−110	−130	−980	160	−97	−160	−71	−71	−110	−77
**Literature**												
L1	−160	−55	−47	−360	−550	−36	−0.42	−0.79	−11	−10	−4.9	−4.1
L2	−270	−130	−120	−560	−670	−64	−2.0	−2.1	−55	−47	−28	−28
L3	−200	−86	−80	−430	−660	−61	−1.9	−2.1	−55	−47	−28	−28
L4	−270	−120	−100	−530	−660	−67	−1.2	−1.0	−20	−20	−15	−12
L5	−200	−80	−71	−410	−650	−64	−1.1	−1.0	−20	−20	−15	−12
L6	−200	−77	−69	−400	−650	−63	−1.0	−0.98	−16	−16	−11	−8.4
L7	−260	−110	−97	−510	−670	−67	−1.1	−0.98	−20	−19	−14	−12
L8	−240	−100	−93	−510	−660	−65	−0.97	−0.93	−16	−16	−10	−8.2
L9	−200	−74	−64	−380	−290	−26	−0.59	−0.68	−16	−15	−10	−8.0
**Posthoc**												
PH1	−260	−100	−85	−510	−860	−83	−1.9	−1.6	−38	−37	−34	−26
PH2	−250	−95	−79	−500	−850	−84	−1.6	−1.4	−1.4	−24	−17	−14
PH3	−300	−110	−97	−610	−1,200	−110	−2.2	−2.0	−31	−31	−20	−16
PH4	−300	−110	−94	−610	−810	−77	−1.2	−1.8	−31	−30	−20	−16
PH5	−470	−200	−170	−920	−1,200	−120	−2.9	−2.4	−52	−51	−45	−35
PH6	−470	−200	−180	−970	−1,400	−130	−1.9	−1.8	−52	−52	−46	−35
PH7	−470	−200	−190	−970	−1,400	−120	−2.1	−2.1	−57	−57	−52	−40
PH8	−600	−250	−220	−1,200	−1,900	−170	−3.2	−2.9	−76	−75	−69	−53
**Nanoindentation**												
N1	−720	−410	−340	−1,600	−1,800	−170	−2.5	−2.4	−33	−31	−17	−14
N2	−400	−210	−180	−820	−880	−90	−1.60	−1.40	−22	−21	−14	−11
N3	−2,000	−1,500	−1,200	−6,000	−6,500	−500	−7.1	−6.3	−57	−50	−22	−19

As expected from the loads applied and bending observed by eye during the experiments, principal strains are highest in the premaxilla (G5), nasals (G7) and lacrimals (G8 and G9), and lowest in the braincase regions (G10–13), despite the latter area being closer to the applied muscle loading. Strain ratio is a measure of the *E_max_* divided by the absolute value of *E_min_* and is useful as it removes the effects of strain magnitudes. Where the strain ratio is between 0.67 and 1.5, shear is the predominant regime. When the strain is greater than 2, or less than 0.5, the regime is either primarily tension or compression respectively (Dzialo et al., 2014). Most of the cranium is in compression, which reflects the bending experienced on the dorsal surface, but there is tension in gauge 7 and a combination of shear and compression in gauge 12 (Table 5).

**Table 5:** Strain ratios (*E_max_*/|*E_min_*|) for the *ex-vivo* experiment and finite element models.

Gauge site	1	2	3	4	5	7	8	9	10	11	12	13
***Ex-vivo***												
Test 1	0.29	−0.32	−0.12	−0.077	−0.0072	5.7	−0.18	0.24	0.048	0.048	0.56	0.27
Test 2	0.17	−0.33	−0.24	0.0078	−0.078	6.3	0.13	0.088	0.085	0.085	0.65	0.40
**Literature**												
L1	0.48	0.80	0.79	0.39	0.47	3.5	1.3	1.8	1.5	1.7	1.1	1.3
L2	0.75	0.68	0.67	0.45	0.27	2.9	1.3	1.7	0.083	0.21	0.77	0.78
L3	0.65	0.82	0.83	0.46	0.27	2.9	1.3	1.7	0.082	0.22	0.77	0.78
L4	0.63	0.62	0.63	0.44	0.27	2.7	1.5	2.3	1.1	1.3	1.2	1.4
L5	0.56	0.75	0.76	0.46	0.27	2.7	1.5	2.3	1.1	1.3	1.2	1.4
L6	0.56	0.75	0.76	0.45	0.27	2.7	1.5	2.2	1.2	1.3	1.2	1.4
L7	0.61	0.67	0.68	0.45	0.26	2.7	1.5	2.3	1.1	1.3	1.2	1.4
L8	0.61	0.67	0.69	0.45	0.26	2.7	1.5	2.2	1.2	1.3	1.2	1.4
L9	0.54	0.70	0.70	0.37	0.48	3.4	0.95	1.7	1.2	1.3	1.2	1.4
**Posthoc**												
PH1	0.57	0.76	1.1	0.46	0.29	2.8	1.5	2.5	0.96	1.2	1.1	1.3
PH2	0.54	0.77	1.1	0.45	0.29	2.7	1.4	2.2	2.2	1.3	1.2	1.4
PH3	0.53	0.79	0.79	0.45	0.32	2.7	1.4	2.2	1.2	1.3	1.2	1.4
PH4	0.51	0.75	0.75	0.39	0.46	2.7	1.3	2.1	1.2	1.3	1.2	1.4
PH5	0.60	0.66	0.66	0.44	0.32	2.8	1.4	2.3	0.99	1.2	1.2	1.4
PH6	0.63	0.75	0.76	0.45	0.22	2.9	1.7	2.5	1.0	1.2	1.2	1.4
PH7	0.64	0.75	0.75	0.45	0.22	3.0	1.6	2.3	0.94	1.2	1.1	1.3
PH8	0.60	0.76	0.77	0.46	0.24	2.9	1.5	2.3	0.97	1.2	1.1	1.4
**Nanoindentation**												
N1	0.79	0.36	0.47	0.42	0.27	2.8	1.5	2.1	1.2	1.4	1.2	1.5
N2	0.77	0.36	0.45	0.42	0.29	2.7	1.4	2.1	1.2	1.3	1.2	1.4
N3	0.79	0.33	0.43	0.41	0.32	3.1	1.3	1.9	1.5	1.7	1.1	1.4

### FEA

#### Material properties from the literature (models L1–L9)

**Figure 4:**
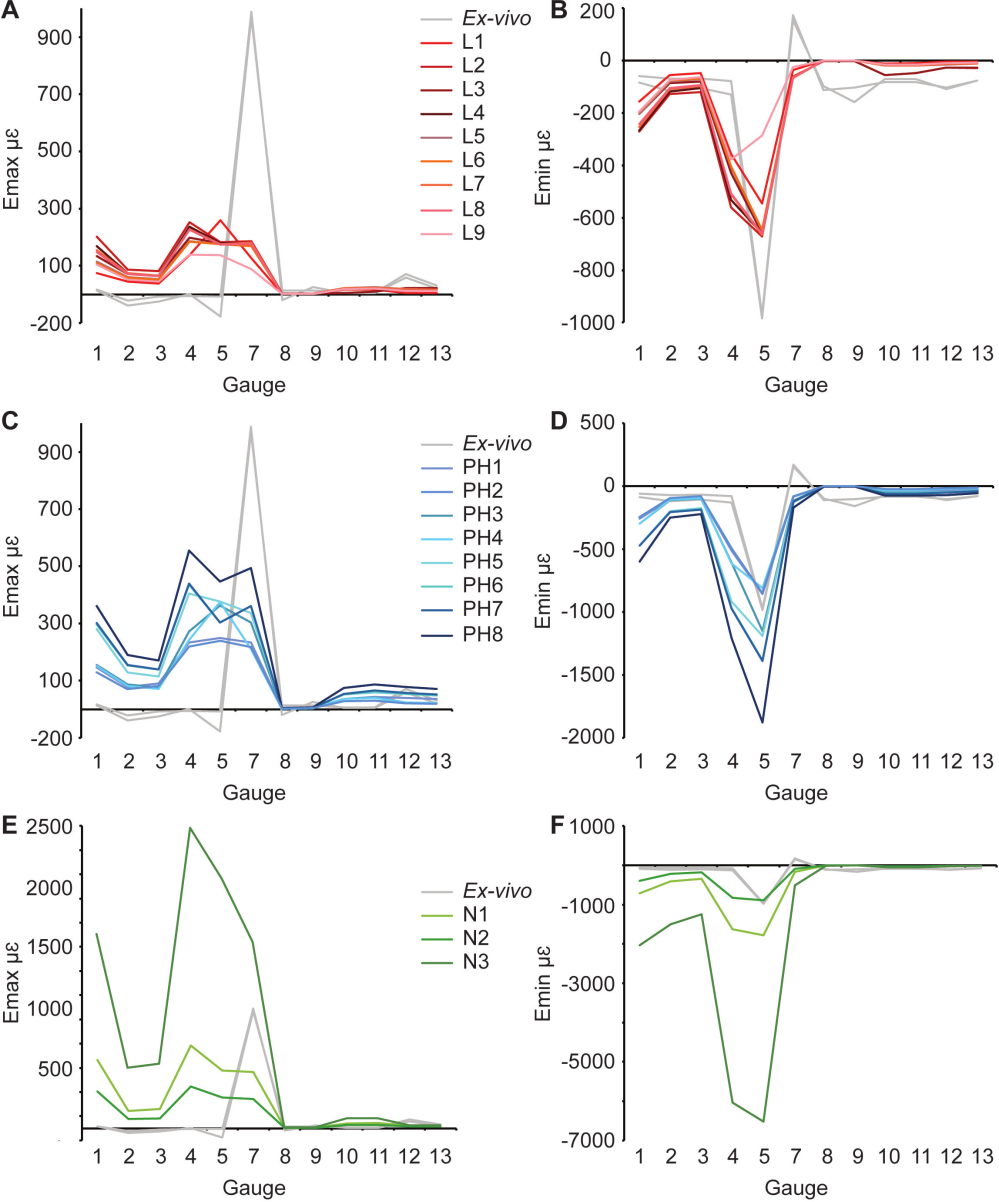
Maximum and minimum principal strains for both *ex-vivo* experiments, and finite element models in microstrain. (A) Maximum, and (B) minimum principal strain for models with material properties from the literature; (C) Maximum, and (D) minimum principal strain for models with posthoc material properties; (E) Maximum, and (F) minimum principal strain for models with material properties from nanoindentation. Material properties for each model are listed in Table 1. Note that both experimental trials are shown.

**Figure 5:**
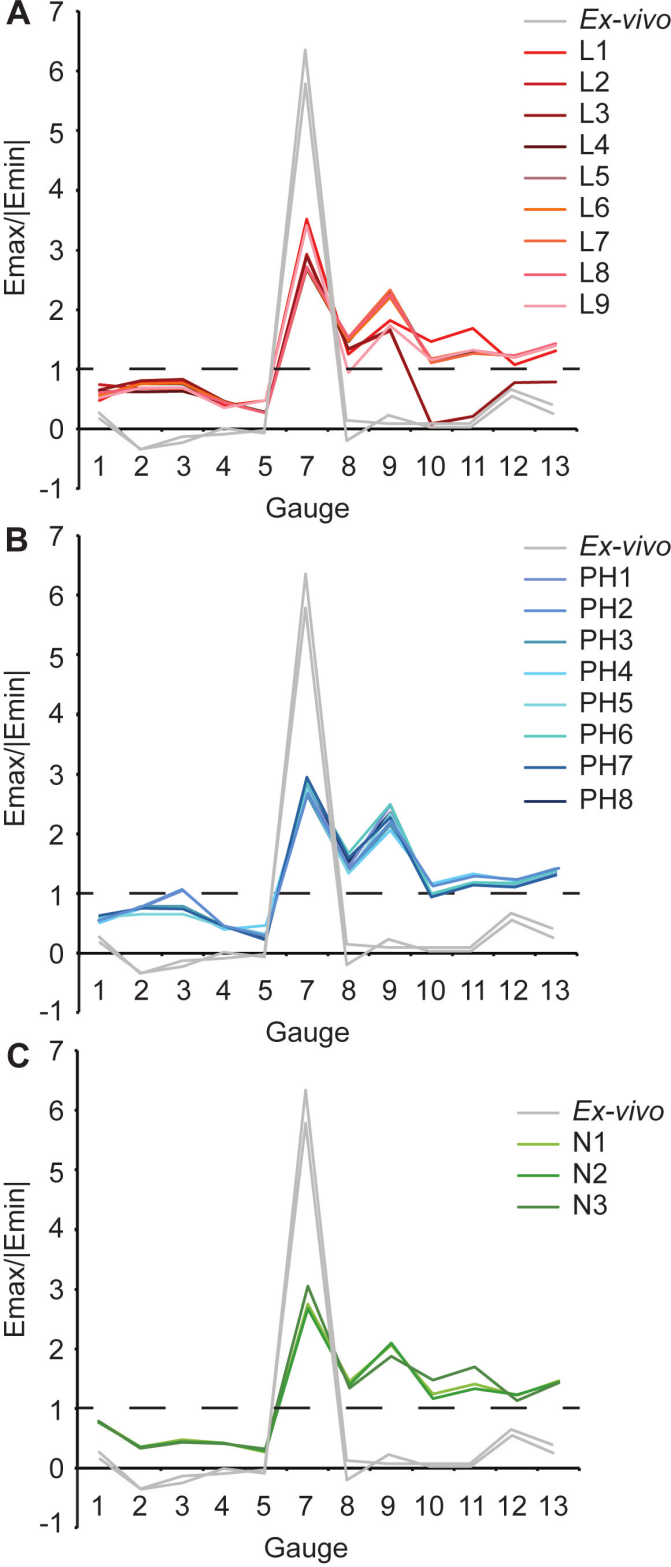
Strain ratios of *ex-vivo* experiment and finite element models. Material properties from (A) the literature; (B) posthoc testing; (C) nanoindentation. Dashed line indicates a strain ratio of 1. Where the strain ratio is between 0.67 and 1.5, shear is the predominant regime. When the strain is greater than 2, or less than 0.5, the regime is either primarily tension or compression, respectively. Note that both experimental trials are shown.

**Figure 6:**
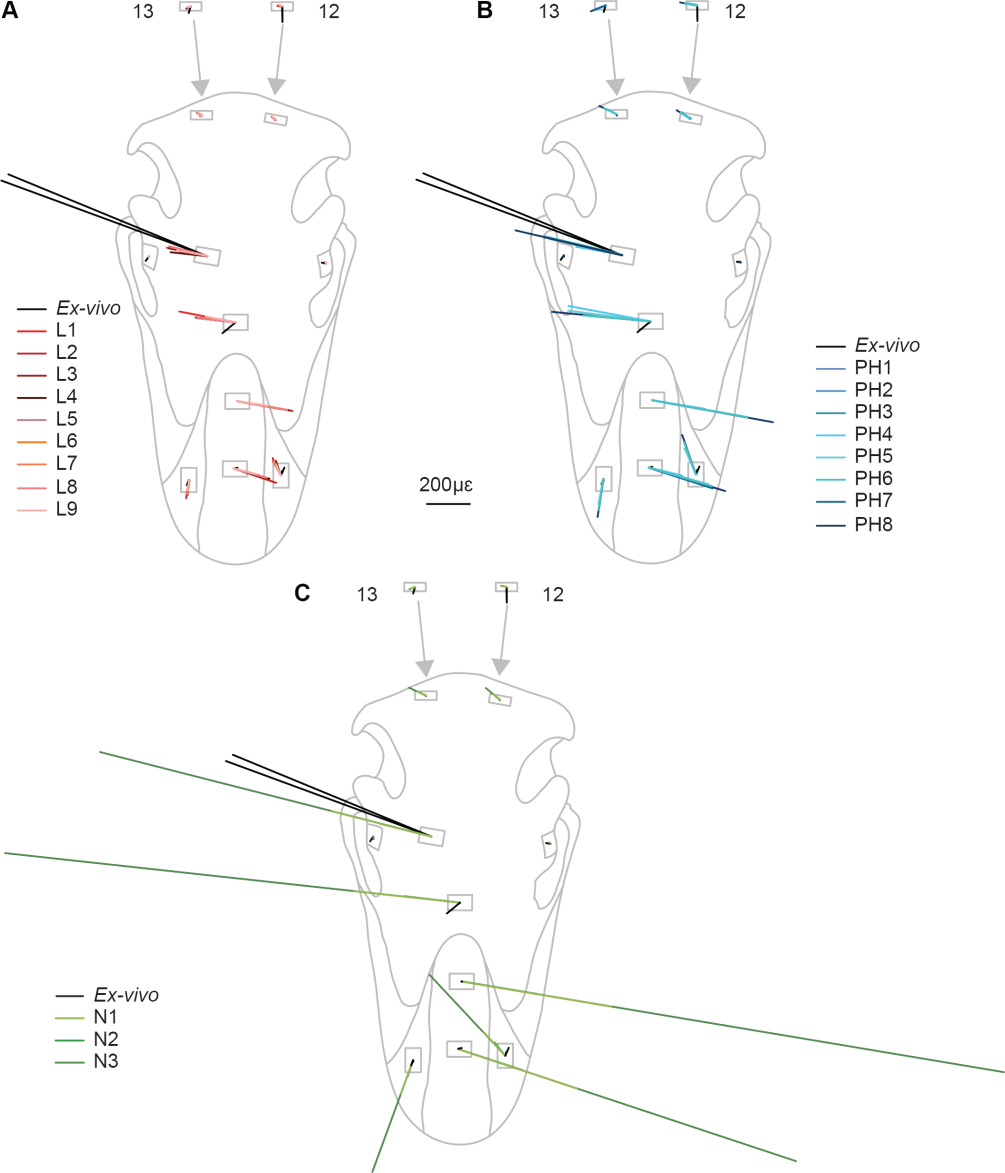
Principal strain orientation and magnitude. Material properties from (A) the literature; (B) posthoc testing; (C) nanoindentation.

Overall there is a high level of similarity between strains in all of the FE models with material properties taken from the literature (Figs. 4A and 4B). There are some similarities between FE tests and the *ex-vivo* experiments but also some key differences. In terms of absolute values of strain magnitude, the areas of closest numerical match are G9–G13, whilst G1–G4 strains follow similar trends to the experimental data but are all offset in *E_max_* with the models being less stiff (i.e., higher strain magnitudes) than the experiment. The FE models represent the peak in *E_min_* at G5 but do not represent the peak in *E_max_* at G7. The FE model strain ratios show the same mode of strain (tension or compression) for G3–G7 (Fig. 5 and Table 5). However, most of the rest of the strain ratios in the models show a combination of shear or either tension or compression, whilst compression is found for the gauges in the *ex-vivo* experiments. Only G4 (on the posterior rhamphotheca) and G7 (on the nasal) have strain orientations within 10°of the orientations recorded in the FE models (Fig. 6 and Table 6). Beyond this there is a range of discrepancy between the experimental strain orientation data and the models (Table 6). Euclidean distance (ED) metrics show that the model with the closest match to the *ex-vivo* data for *E_max_*, *E_min_* and strain orientations was L3 but for strain ratios and L2 provided the best overall match (Table 7). Regressions show there is no significant relationship between the experimental data and the averaged data for the FE models (i.e., values for L1–L9 averaged) for *E_max_* (Figs. S10–S11), and strain orientations (Fig. S16). There is a significant correlation between the experimental data and the averaged data for *E_min_* (Fig. S12) and strain ratios (Fig. S14), but when possible outliers are removed (see Supplemental Information) the correlations in *E_min_* (Fig. S13) and strain ratios (Fig. S15) become non-significant. The Euclidean distances also change if the possible outliers are removed, with L9 performing best for strain magnitudes instead of L3 (Table S3).

**Table 6:** Principal strain orientations from grid one direction on the gauge, in degrees.

Gauge site	1	2	3	4	5	7	8	9	10	11	12	13
***Ex-vivo***												
Test 1	34	60	17	55	−10	55	−6.5	48	16	−59	−46	−29
Test 2	48	68	78	59	−4.5	57	−6.0	45	12	−59	−46	−29
**Literature**												
L1	57	23	53	54	56	45	38	37	78	70	55	19
L2	64	20	55	55	52	47	19	17	43	50	54	17
L3	62	26	52	55	51	47	18	18	44	51	54	17
L4	61	15	57	55	51	49	28	1.0	71	70	56	25
L5	60	23	54	55	51	49	27	2.5	71	70	56	25
L6	60	21	54	55	51	49	26	3.7	72	69	56	25
L7	61	18	55	55	51	49	25	3.5	71	70	56	25
L8	61	16	56	55	51	49	23	5.1	72	69	56	25
L9	59	20	55	54	56	45	43	39	72	69	56	25
**Posthoc**												
PH1	60	26	53	55	52	47	28	11	67	70	56	24
PH2	59	23	54	55	52	49	30	4.0	72	69	55	25
PH3	59	24	53	55	52	49	31	7.3	72	69	56	25
PH4	59	23	54	54	56	45	40	37	72	69	56	25
PH5	61	21	55	55	52	47	32	11	68	70	56	24
PH6	61	25	53	55	50	49	11	3.6	68	70	56	24
PH7	63	25	53	55	50	48	8.4	5.0	67	70	56	23
PH8	62	26	53	55	51	48	16	4.8	67	70	56	23
**Nanoindentation**												
N1	63	2.6	64	55	51	49	22	3.7	75	70	56	24
N2	63	6.0	63	55	52	49	28	1.5	73	70	56	25
N3	63	1.9	65	55	51	49	21	3.7	75	70	56	24

**Table 7:** Euclidean distances from *ex-vivo* experimental measures to FE model data. Data in bold are the closest to the experimental data for each specific metric.

	*E_max_*	*E_min_*	Strain ratio	Orientation
**Literature**				
L1	922	591	4.39	203
L2	893	657	**4.04**	180
L3	**873**	**556**	4.10	**179**
L4	890	648	4.89	207
L5	877	561	4.93	204
L6	874	557	4.91	204
L7	886	627	4.90	205
L8	884	627	4.89	205
L9	933	805	4.09	205
**Posthoc**				
PH1	864	559	4.96	201
PH2	**860**	**560**	5.28	204
PH3	864	690	4.90	204
PH4	934	660	4.82	204
PH5	931	1,003	4.76	203
PH6	917	1,108	4.92	200
PH7	920	1,106	**4.68**	**200**
PH8	1,002	1,587	4.75	200
**Nanoindentation**				
N1	1,171	1,912	4.75	**210**
N2	**932**	**873**	4.74	210
N3	3,773	8,526	**4.55**	210

#### Posthoc values (models PH1–8)

For *E_max_*, the results are similar to those for the literature models. Gauges 1–4 show a similar pattern of *E_max_* values, but again, FE model magnitudes are greater than the *ex-vivo* data (Fig. 4C, Table 3). Some of the models (PH6–8) replicate peaks at G7, but do not achieve the magnitude of strain recorded in the *ex-vivo* data (Fig. 4C). Other models do not replicate this peak. For *E_min_* (Table 4), the strain magnitudes at sites G2 and G3 in the FE models closely match the *ex-vivo* experiment but strains in G1 and G4 are higher in the FE models than experimentally (Fig. 4). The peak at G5 in the experimental data is replicated in the FE models, as is the decline in *E_min_* magnitude in the more posterior gauges G8–G13.

The *ex-vivo* experimental data are broadly similar to the FE models with respect to strain ratios from G3–7 (Table 5). However, most of the rest of the strain ratios in the models show a combination of shear or either tension or compression, whilst compression is found for the gauges in the *ex-vivo* experiments. Again the strain orientations in all of the models (PH1–PH8) vary in how much they match experimental strain orientations, with only G4 and G7 falling within 10° of the *ex-vivo* values (Fig. 6 and Table 6). The Euclidean distances show that PH2 is most similar to the *ex-vivo* data for strain magnitudes whilst PH7 best matches the strain orientations and strain ratios (Table 7). The regressions show there is no significant relationship between the experimental data and the averaged data for the FE models for *E_max_* (Figs. S10 and S11), and the strain orientations (Fig. S16). There is a significant correlation between the experimental data and the averaged data for *E_min_* (Fig. S12) and strain ratios (Fig. S14), but when possible outliers are removed the correlations in *E_min_* (Fig. S13) and strain ratios (Fig. S15) become non-significant. The Euclidean distances also change if the possible outliers are removed, with PH4 becoming best for strain orientations (Table S3).

#### Nanoindentation values (models N1–N3)

For *E_max_*, all three FE models fail to replicate the peak in strain magnitude at G7. Low strain magnitudes are observed in G8–G13 in both the experimental and the model data, but the FE models record much higher strains at G1–G4 than either the literature (L1–L9) or posthoc (PH1–8) datasets (Table 3 and Fig. 4E). In particular, model N3 produces strains that are far higher than those seen in any of the other FE models or the *ex-vivo* data. (Fig. 4E). The peak in experimental *E_min_* at G5 is replicated in the FE models (Table 4) with the maximum material properties model (N2) most closely matching that of the *ex-vivo* data (Fig. 4F). As with the other models (L1–9 and PH1–8), the strain ratios match the experiments in terms of compression or tension for G2–7 (Fig. 5C and Table 5). However, most of the rest of the strain ratios in the models show a combination of shear or either tension or compression, whilst compression is found for the gauges in the *ex-vivo* experiments. The strain orientations for N1–3 are quite disparate from those found in the experiment, except for G4 and G7 (Fig. 6 and Table 6). The regressions show there is no significant relationship between the experimental data and any of the FE models for *E_max_* (Figs. S10 and S11), and the average data for strain orientations (Fig. S16). There is a significant correlation between the experimental data and the average FE data for *E_min_* (Fig. S12) and strain ratios (Fig. S14), but when possible outliers are removed the correlations in *E_min_* (Fig. S13) and strain ratios (Fig. S15) become non-significant. The Euclidean distances also change if the possible outliers are removed, with N2 closer to the experimental average for strain orientations than N3 (Table S3).

## Sutures

**Figure 7:**
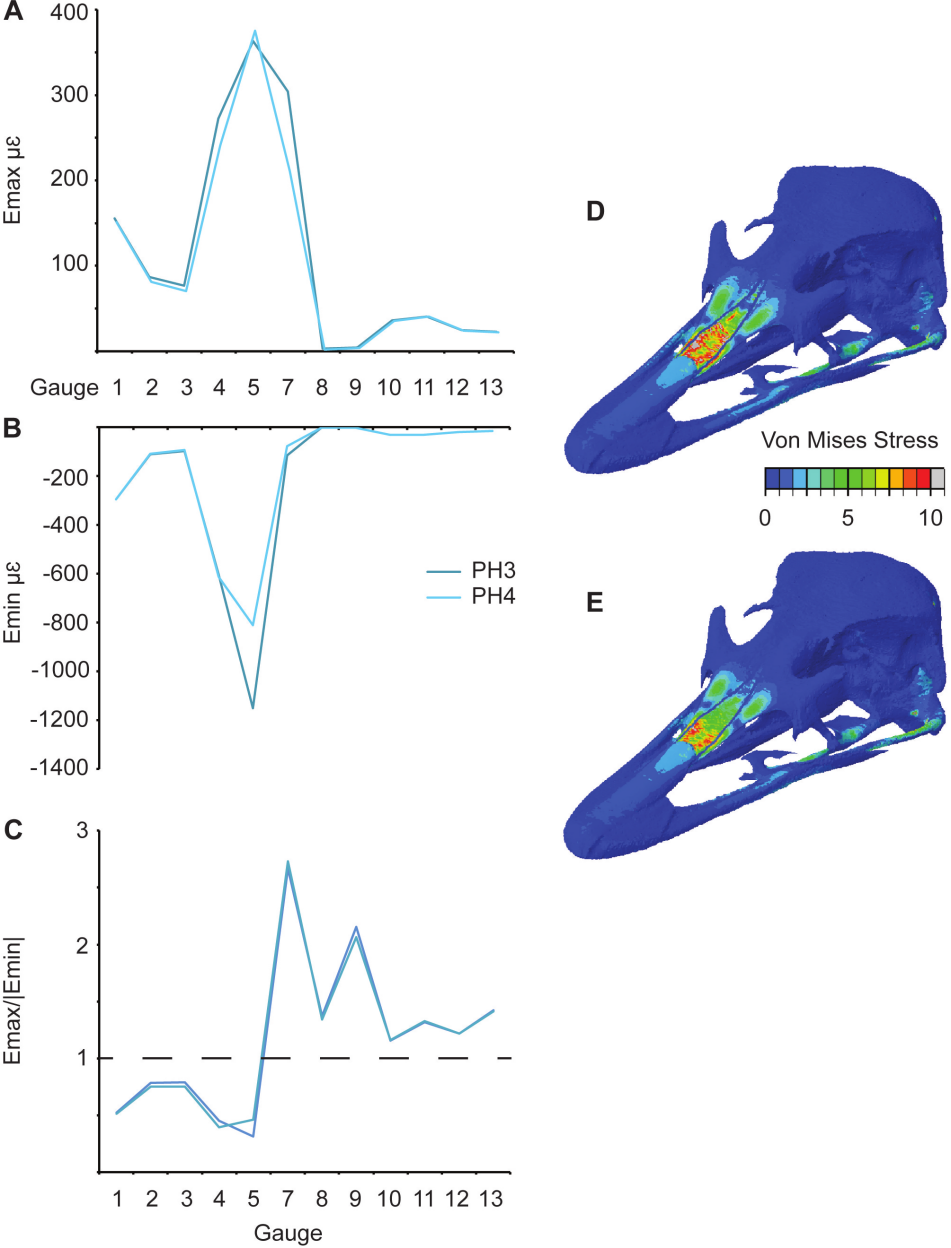
Comparison of results between models (PH3 and PH4) with different suture properties (see Table 1). (A) Maximum and (B) minimum principal strain, (C) strain ratio, and Von Mises (in MPa) stress for (D) PH3 and (E) PH4.

Three pairs of models are equivalent except for their sutural properties (Table 1): L6 and L9; PH3 and PH4 (Fig. 7); and PH5 and PH6. In general an increase in Young’s modulus of the sutures leads to a decrease in the overall maximum principal strains (Fig. 7A and Table 3). When comparing PH5 and PH6, there are lower strains in G7–G9 for the model with the lower sutural Young’s modulus. The models that possess the lower sutural Young’s modulus have the largest peak minimum principal strain values (Fig. 7B and Table 4). The strain ratios are congruent with respect to compression or tension in the models, and only small variations in numerical values are seen (Fig. 7C and Table 5). Strain orientations are generally unaffected by changing sutural properties with only small variations in values except in G8 and G9 which possess much smaller angles in the models with lower Young’s moduli for all pairings (Table 6). Visual observation of the FE models show that Von Mises stress is higher in models with lower sutural Young’s moduli (Figs. 7D and 7E) whilst the presence of sutures leads to a sharp drop in stress between the premaxilla and nasals and in the nasal region (Figs. 7D and 7E).

**Figure 8:**
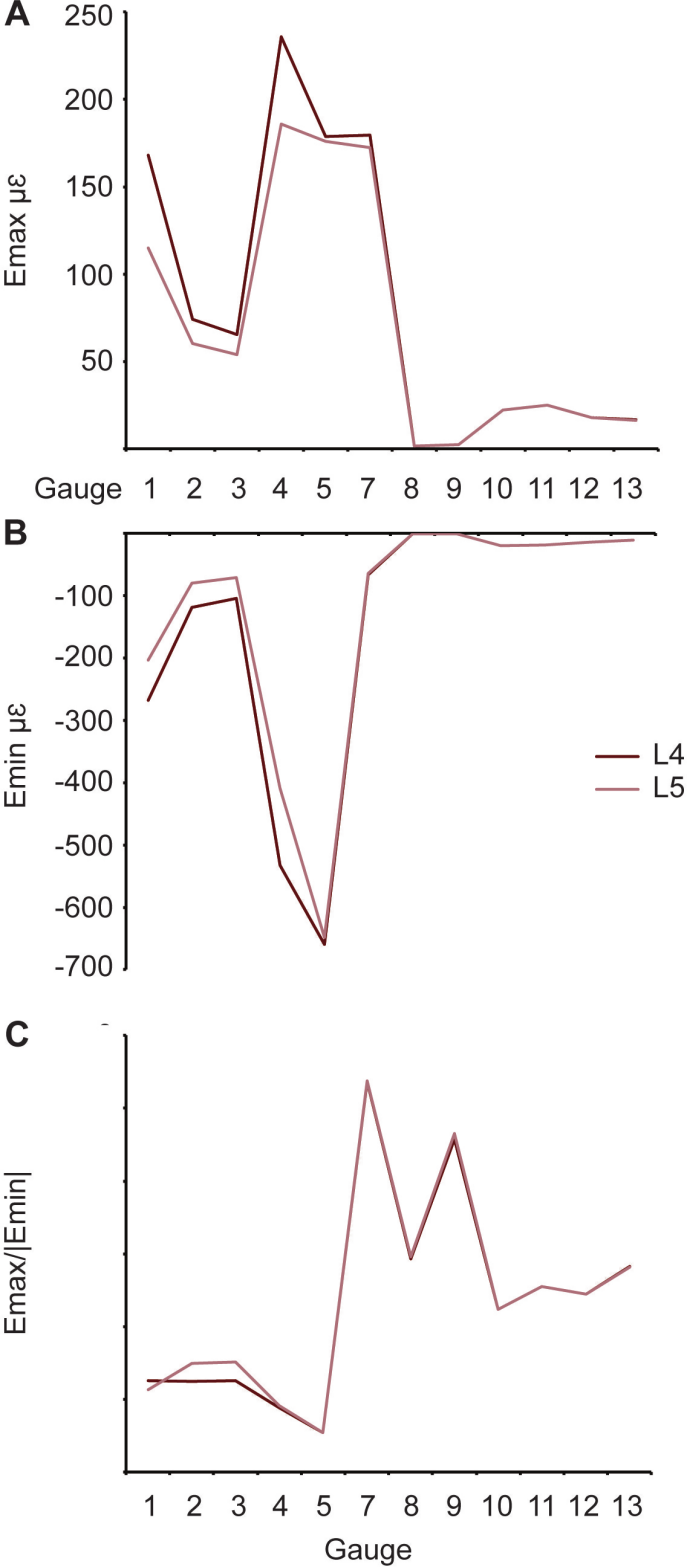
Comparison of results between models (L4 and L5) with different rhamphotheca properties (see Table 1). (A) Maximum and (B) minimum principal strain and (C) strain ratio.

### Rhamphotheca

One pair of models (L4 and L6) possessed the same material properties except for the rhamphotheca (Table 1). As with the sutural tests, an increase in Young’s modulus of keratin leads to a decrease in the overall maximum principal strains within in the model (Fig. 8A and Table 3). For minimum principal strains, the peak values are found in the model with the lowest Young’s modulus (Fig. 8B and Table 4). The strain ratios are approximately the same between the models with differing properties, and there are no differences with respect to compression or tension at the gauge sites (Fig. 8C and Table 5). Strain orientations are all very similar between both models (Table 6).

## Discussion

There is some difference in absolute magnitudes of strain between the two experimental trials, but the data are not statistically different. Residual loading of the cranium and thermal drift were accounted for in the experimental set up and it is unlikely either is responsible for the discrepancy between strain magnitudes in the two trials. Slight movement of the specimen on the testing rig could have influenced the results. Also, desiccation of the specimen during the trials may have been a factor, even though the cranium was kept as moist as possible with a glycerol-water solution. In addition, due to only being able to record data from four gauges at any time means that there is probably some variability in the loading the cranium is undergoing during each test.

The validity of the finite element models, with respect to how accurately they represent experimental strains, is highly variable depending on both the metric under consideration, and the anatomical region of interest and for nearly all metrics falls beyond the limits of error assumed in previous cranial validation studies (e.g., Ross et al., 2005; Metzger, Daniel & Ross, 2005; Bright & Rayfield, 2011a). The FE models tend to replicate the location of peak strains in the experimental cranium, but not perfectly, particularly with respect to maximum principal strains (G5 and G7). At strain peaks the FE models are too stiff, but at other, lower strain locations, the FE models are less stiff than the experimental data. This finding is, in part, contrary to some other validation studies on different taxa that tend to find the FE models are overall too stiff compared to experimental data (e.g., Metzger, Daniel & Ross, 2005; Bright & Rayfield, 2011a; Porro et al., 2013). This phenomenon could be attributed to improper representation of Young’s moduli heterogeneity and anisotropy in models (Berthaume et al., 2012), improper representation of cortical bone or rhamphotheca thickness (Anderson et al., 2005; Gröning, Fagan & O’Higgins, 2013), improper representation of cranial sutures (in terms of both material and geometry; Moazen, Costantini & Bruner, 2013), or some combination of these factors.

In terms of replicating the overall mechanical behaviour of the cranium, the strain ratios generally record similar modes of strain (tension or compression) in the rostrum (gauges 1-7) but not in the neurocranium (gauges 8-13). It is unclear whether this is a feature of the single cranium tested or failure to accurately model the synovial joints (which were modelled as sutures) separating the palate from the neurocranium. Another variable that needs further consideration is the novel method of loading the cranium using the artificial tendon. Whilst the method attempts to mitigate issues of either using preserved muscle fibres (Kupczik et al., 2007) or metal screwed into the cranium (e.g., Bright & Rayfield, 2011a), it was ultimately necessary to screw the fibreglass into the cranium to maintain the tendon/cranium interface. We are not certain how the load was affected by the screws as this was not explicitly tested; however, previous studies utilising screws in the cranium (which also did not replicate the holes created in the model) managed to successfully replicate *ex-vivo* strain patterns in the finite element model (Bright & Rayfield, 2011a; Bright & Gröning, 2011).

Furthermore, strain orientations between the experiment and the models are within 10° in relatively few locations on the cranium, with other sites showing variable degrees of correlation. Interestingly, no single model produces the lowest Euclidean Distances for all four metrics compared to the experimental values. Overall, the best estimates of *E_max_* and *E_min_* are given by models L9 and PH2 respectively, and the best estimates of strain ratio and orientation are given by model L3. However, it must be noted that each of these models still fail to replicate individual gauge strains in certain locations (e.g., G12 in L9 is an order of magnitude different from the experimental data) despite being the best overall match.

The regression analyses (see Supplemental Information) show that although experimental performance is consistent, there is no correlation between experimental data and FE models for maximum principal strain and strain orientation. The lack of correlation may in part be due to some regions of the cranium showing a good match between experimental and FE data, and other regions showing a poor match. There are significant correlations between the experimental data and FE models for minimum principal strain and strain ratios. *R*-squared values are variable, and heavily influenced by the presence of potentially outlying data points (G7 for *E_max_* and strain ratio, and G5 for *E_min_*). Removal of these data points makes relationships non-significant, but whether or not these outliers should be included for consideration is unclear. It is possible that G7 may have been recording erroneously high strains, but the values obtained between experimental trials were very similar (and less variable than some other gauge sites) so this seems unlikely. Indeed, the strain ratio peaks are replicated (albeit with lower values) in the FE models. Visual observation of the experiment confirmed that the cranium undergoes bending in the vicinity of these gauges (see also Gussekloo & Bout, 2005) hence these values may not be erroneous. That the data are relatively well replicated in both the experiments and FE models and this suggests that G7 and G5 are providing a reliable signal and should be included in considerations of model fit (see Supplemental Information for further information).

As our experimental data was obtained from a single specimen, it is not possible to tell whether these high strains are in fact typical for ostriches, or whether gauge performance was unusual. The current investigation sought to compare a specimen-specific FE-model to experimental data without the confounding effects of intraspecific variation (such as differences in gross geometry, material properties, amount of cancellous bone or other microstructural or mineralization effects), and there are limitations to the single specimen test approach; despite every effort being taken to maintain the hydration of the specimen (and therefore the tissues), dehydration undoubtedly occurred and this may have affected particular tissues (e.g., sutures) more than others (e.g., cortical bone). The relatively thin cranial bones of birds and limited soft tissue still adhering to the specimen may have been a factor. Dehydration may be responsible for variation between the two experimental trials and may have contributed to differences between the experimental and FE model data (Soons et al., 2012a). Both the effects of intraspecific variation, and of specimen hydration, should be investigated further in the future.

During the experimental trials, the strains were collected by gauges. This method has limitations, as gauges can suffer failure (as seen with G6). In addition, gauges work best on flat surfaces, yet in biological structures such as a cranium, flat surfaces are difficult to identify. Other methods for investigating strain magnitudes and orientations, such as digital speckle pattern interferometry (DSPI; e.g., Bright & Gröning, 2011; Soons et al., 2012c) and digital volume correlation (DVC; Bay et al., 1999), allow for non-contact sampling of the objects and may be preferred in situations where strain gauges cannot be used (e.g., curved surfaces or small specimens), although the high costs and low signal-to-noise ratio associated with these methods may be prohibitive. It would nevertheless be interesting to validate the strain gauge strains with strains obtained from one of the other methods.

### Material property considerations

Within the models, each material is modelled as isotropic. It is unlikely that many biological bony structures are isotropic throughout, and probably have variable material properties and anisotropy depending on location (e.g., Zapata et al., 2010). This is supported by our nanoindentation data documenting the high variability in material property standard deviations across just an individual test location, and between different sites on the rostrum (Table 2). The use of isotropic and internally homogeneous material properties for bone, keratin and sutures may explain the lack of statistically significant correlation between our *ex-vivo* results and FE models, despite the fact that the models are broadly deforming as expected. Furthermore, the methods used here may fail to accurately model the complex bone/beak/suture interfaces. The use of anisotropic material properties may have improved model data fit, but this was beyond the scope of this study and would require more extensive nanoindentation sampling across the entirety of the cranium. It is possible that such concerns could be mitigated by restricting future validation tests to smaller regions of presumably similar function, for instance either the facial region (e.g., Soons et al., 2012a) or the braincase, particularly when model performance seems to be so affected by anatomical location, as seen here and in previous validation studies on pigs (Bright, 2012) and ostrich mandibles (Rayfield, 2011). There is also a chance that there is intraspecific variability with regards to the material properties; a different ostrich was used for the nanoindentation than for the FE modelling. In this case the animals were from the same stock and culled at the same age, so these effects were minimised as best possible.

### Effects of sutures

The *ex-vivo* experimental data follows the results of many previous experimental studies showing that the strain environment can change dramatically between bones separated by a suture (Jaslow, 1990; Herring & Mucci, 1991; Jaslow & Biewener, 1995; Herring & Teng, 2000; Bright & Gröning, 2011; Bright, 2012). The maximum (Table 3) and minimum principal strains (Table 4), strain ratios (Table 5) and strain orientations (Table 6) change markedly between G5, G7 and G9 which are located on the premaxilla, right nasal and right lacrimal and each separated by a suture (Fig. 3). There is a rapid drop in Von Mises stress across the sutures in the rostra, particularly between the high stress area of the premaxilla and the lower stressed nasals (Fig. 7).

The presence of sutures and variation in their elastic properties is known to effect overall FE model stress in certain taxa (Reed et al., 2011; Bright, 2012; Moazen, Costantini & Bruner, 2013), but not in all (e.g., macaques—Wang et al., 2010). The sutural effects may vary depending on the taxon, volume of sutures or even the method used to model the sutures. The sutures in this study were modelled as tetrahedral elements with lower Young’s moduli than the surrounding bone, as for previous analyses (Moazen et al., 2009; Wang et al., 2010; Reed et al., 2011; Bright & Gröning, 2011; Bright, 2012). The presence of sutures in our models affected all measured metrics such that the models with sutures were better at replicating the experimental minimum principal strains, strain ratios and orientations than those that did not possess sutures. However, models L1 and L9, which do not have sutures, have values on each extreme for the Euclidean distances: L1 is very different in maximum principal strain to the *ex-vivo* data, and L9 is the most similar to the experimental maximum principal strain. Sutures alone therefore do not rectify all of the differences seen between the *ex-vivo* experimental values and the FE models.

Ostriches possess cranial sutures between all of their cranial bones, and through ontogeny many of these fuse. This fusion is not uniform; those across the braincase fuse first and those in the high strain regions of the premaxilla/nasal/lacrimal region stay unfused into adulthood (AR Cuff, pers. obs., 2011). This, combined with the experimental data, suggests that the sutures are playing an active role in strain mitigation; regions of low strain such as the braincase do not need sutural flexibility so they fuse, whilst sutures in regions of higher strain such as the beak remain patent and maintain a functional influence.

### Effects of the rhamphotheca

The rhamphotheca is a complex, composite, keratinous structure that covers portions of the bony rostra in birds. Its extent varies between palaeognaths and neognaths, and within each group, but in all taxa its primary function is feeding. Three previous studies have tested the ability to model the beak using FEA in toucans (Seki, Mackey & Meyers, 2012), and finches (Soons et al., 2012b; Soons et al., 2012c). These studies focus only on the rostral regions containing the beak, but show that FE models can accurately replicate strain patterns across the beak. Indeed, they suggest that the keratinous rhamphotheca surrounding the bone (which may well be heavily trabeculated in the beak region) protects the bone from buckling during impact loadings and removal of the rhamphotheca leads to failure of the bone under equivalent forces (Soons et al., 2012b).

The rhamphotheca in ostriches was modelled as a homogenous structure overlying the bone with a lower Young’s modulus than the cortical bone it surrounds. The experimental data shows that the gauges on the dorsal surface of the rhamphotheca are less strained than the nearest gauges directly on the bone, particularly in minimum principal strain. Increasing the Young’s modulus of the keratin decreases the strains experienced both on the rhamphotheca and throughout the model. The mechanical influence of a rhamphotheca would be especially important across the areas of high strain and thin bone of the rostra and buccal margins, as was found in the beaks of herbivorous theropod dinosaurs (Lautenschlager et al., 2013). In ostriches, the rhamphotheca extends over the premaxilla, and the ventrolateral edges of the maxillae and anteriormost jugals. There may also be a gradational transition of keratinisation from the rhamphotheca *sensu stricto* to the skin (Genbrugge et al., 2012). This phenomenon was not modelled here, but warrants further investigation. Comparison to material property data obtained from previous nanoindentation studies on various areas of the beak suggest that ostrich beak keratin may have a far lower Young’s modulus (by up to a factor of two) than that seen in other birds (Seki, Bodde & Meyers, 2010; Soons et al., 2012a). It should be noted that the areas tested in our nanoindentation study were the internal regions of the rhamphotheca, which may have a far lower Young’s moduli than the tougher outermost layer. Further modelling will be required to test the overall effect this may have on strain patterns and orientations in FE models.

### Mechanical behaviour of the beak versus the braincase

In general, the experimental and modelled strain ratios match more closely in the beak than they do in the braincase. This may be because the braincase is closer to the constraints emplaced on the quadrate. Yet, strain magnitudes in the braincase region are low, meaning a small change in strain magnitude could switch the braincase from experiencing primarily compression to primarily tension, or vice versa. These very different strain signals point to a functional signal represented in this data, suggesting a modular biomechanical response between the functions of the beak and the cranium.

## Conclusion

Using new data from nanoindentation studies and multiple material properties drawn from the literature, it is possible to test the effectiveness of the FE method on modelling the mechanical behaviour of an avian cranium. We find that it may be important to model the rhamphotheca and sutures separately, with both affecting strain patterns and magnitudes. Whilst the material properties used in this study fail to replicate peak strain magnitudes on the bone or on the beak, they do approximate absolute values in low strain areas, and demonstrate broadly accurate strain patterns reflecting the location of peak strains (although not exclusively). However, the mode of strain in the neurocranial region (tension versus compression) is often not replicated in the FE model. Our FE models replicate some of the strain orientation measurements across the cranium as in previous studies but the majority do not match closely. These differences warrant further study with different methods to further investigate the cause of such discrepancy between FE model and experimental strain data. In spite of such limitations, our results do demonstrate that the overall mechanical behaviour of the cranium illustrates a highly strained rostrum yet low strains across the neurocranium, potentially demonstrating functional modules in the beak versus the braincase.

## Supplemental Information

10.7717/peerj.1294/supp-1 Supplemental Information 1 3D pdf of the ostrich model The grey material is cortical bone, the red rectangles are membrane elements that mirror the strain gauges. Gauge 6 was non-functional so was not included in the model. The blue lines are sutures, and the yellow material is the keratinous rhamphotheca. The trabecular bone is a light yellow under the cortical bone.

10.7717/peerj.1294/supp-2 Supplemental Information 2 Supplementary information

## Additional Information and Declarations

### Data Availability

The following information was supplied regarding data availability: All raw data has been included in the manuscript and in the Supplemental Information, with the exception of the CT scans and the full model. The CT scans are being used for ongoing research, so cannot be published at this time. The full model is in a format that is only usable by a single program. The geometry has been converted into a 3D PDF for viewing (see Supplemental Information). A link to an example model file can be sent by request to andrew.cuff@ucl.ac.uk.
