# Supplementary figures and images for: Validation experiments on finite element models of an ostrich (Struthio camelus) cranium
Source: PeerJ. 2015 Oct 13;3:e1294. doi: 10.7717/peerj.1294 (PMC4614885; doi:10.7717/peerj.1294)

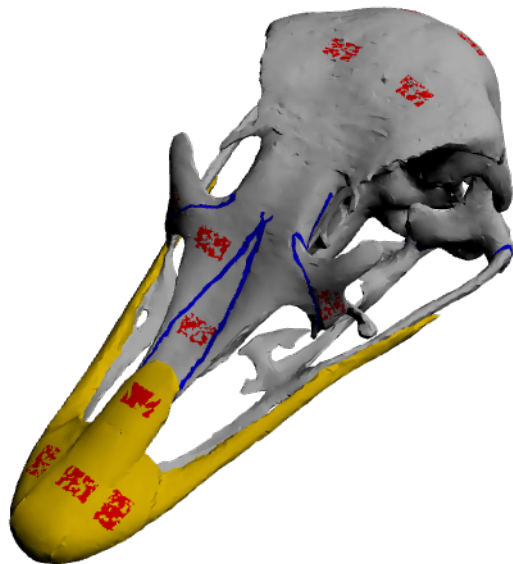

Supplement: Supplemental Information 1 — The grey material is cortical bone, the red rectangles are membrane elements that mirror the strain gauges. Gauge 6 was non-functional so was not included in the model. The blue lines are sutures, and the yellow material is the keratinous rhamphotheca. The trabecular bone is a light yellow under the cortical bone. [file peerj-03-1294-s001.pdf]
