# Supplementary material for: Validation experiments on finite element models of an ostrich (Struthio camelus) cranium
Source: PeerJ. 2015 Oct 13;3:e1294. doi: 10.7717/peerj.1294 (PMC4614885; doi:10.7717/peerj.1294)
Supplement: Supplemental Information 2 [file peerj-03-1294-s002.docx]

**(9) Supplementary information**

**Convergence**

All of the models were loaded with the same forces as those of the *ex*-*vivo* experiment, with homogeneous material properties across the skull, sutures and rhamphotheca (E = 7GPa, v = 0.35 after Rayfield, 2011).

Table S1. E_max_ for the convergence testing of the ostrich model. All values reported in strain (ɛ).

|  | | Tet 4 | | | | | Tet10 | | | | |
| --- | --- | --- | --- | --- | --- | --- | --- | --- | --- | --- | --- |
| Number of elements | | 1333804 | 1518437 | 1631592 | 1708994 | 1749533 | 1333804 | 1518437 | 1631592 | 1708994 | 1749533 |
| Gauge number | 1 | 1.03E-04 | 1.03E-04 | 1.02E-04 | 1.02E-04 | 1.04E-04 | 1.16E-04 | 1.17E-04 | 1.16E-04 | 1.14E-04 | 1.17E-04 |
|  | 2 | 8.07E-05 | 8.04E-05 | 7.50E-05 | 7.51E-05 | 7.02E-05 | 7.73E-05 | 8.08E-05 | 7.63E-05 | 7.79E-05 | 7.17E-05 |
|  | 3 | 9.14E-05 | 8.95E-05 | 9.32E-05 | 8.98E-05 | 1.03E-04 | 1.21E-04 | 1.13E-04 | 1.15E-04 | 1.05E-04 | 1.26E-04 |
|  | 4 | 1.16E-04 | 1.17E-04 | 1.14E-04 | 1.19E-04 | 1.17E-04 | 1.34E-04 | 1.36E-04 | 1.31E-04 | 1.35E-04 | 1.35E-04 |
|  | 5 | 2.76E-04 | 2.77E-04 | 2.80E-04 | 2.88E-04 | 2.80E-04 | 3.75E-04 | 3.73E-04 | 3.66E-04 | 3.78E-04 | 3.77E-04 |
|  | 7 | 1.53E-04 | 1.70E-04 | 1.63E-04 | 1.80E-04 | 1.80E-04 | 2.49E-04 | 2.76E-04 | 2.54E-04 | 2.73E-04 | 2.77E-04 |
|  | 8 | 2.42E-05 | 2.45E-05 | 2.49E-05 | 2.80E-05 | 2.51E-05 | 2.87E-05 | 2.91E-05 | 2.94E-05 | 2.91E-05 | 2.82E-05 |
|  | 9 | 2.21E-05 | 2.14E-05 | 2.13E-05 | 2.37E-05 | 2.12E-05 | 2.63E-05 | 2.55E-05 | 2.52E-05 | 2.79E-05 | 2.54E-05 |
|  | 10 | 5.78E-06 | 6.49E-06 | 5.16E-06 | 4.47E-06 | 6.94E-06 | 5.77E-06 | 6.47E-06 | 5.11E-06 | 4.32E-06 | 6.91E-06 |
|  | 11 | 1.39E-05 | 1.38E-05 | 1.32E-05 | 1.36E-05 | 1.42E-05 | 1.40E-05 | 1.40E-05 | 1.35E-05 | 1.37E-05 | 1.44E-05 |
|  | 12 | 7.71E-06 | 7.83E-06 | 7.13E-06 | 7.23E-06 | 7.17E-06 | 7.48E-06 | 7.66E-06 | 6.96E-06 | 7.07E-06 | 6.96E-06 |
|  | 13 | 5.65E-06 | 5.77E-06 | 6.07E-06 | 6.11E-06 | 5.28E-06 | 5.44E-06 | 5.56E-06 | 5.84E-06 | 5.93E-06 | 4.99E-06 |

Figure S1. E_max_ for the convergence testing of the ostrich model. Each model is represented by a different column of coloured dots. Each colour is representative of a single gauge location. The line separates tet4 from tet10 models.


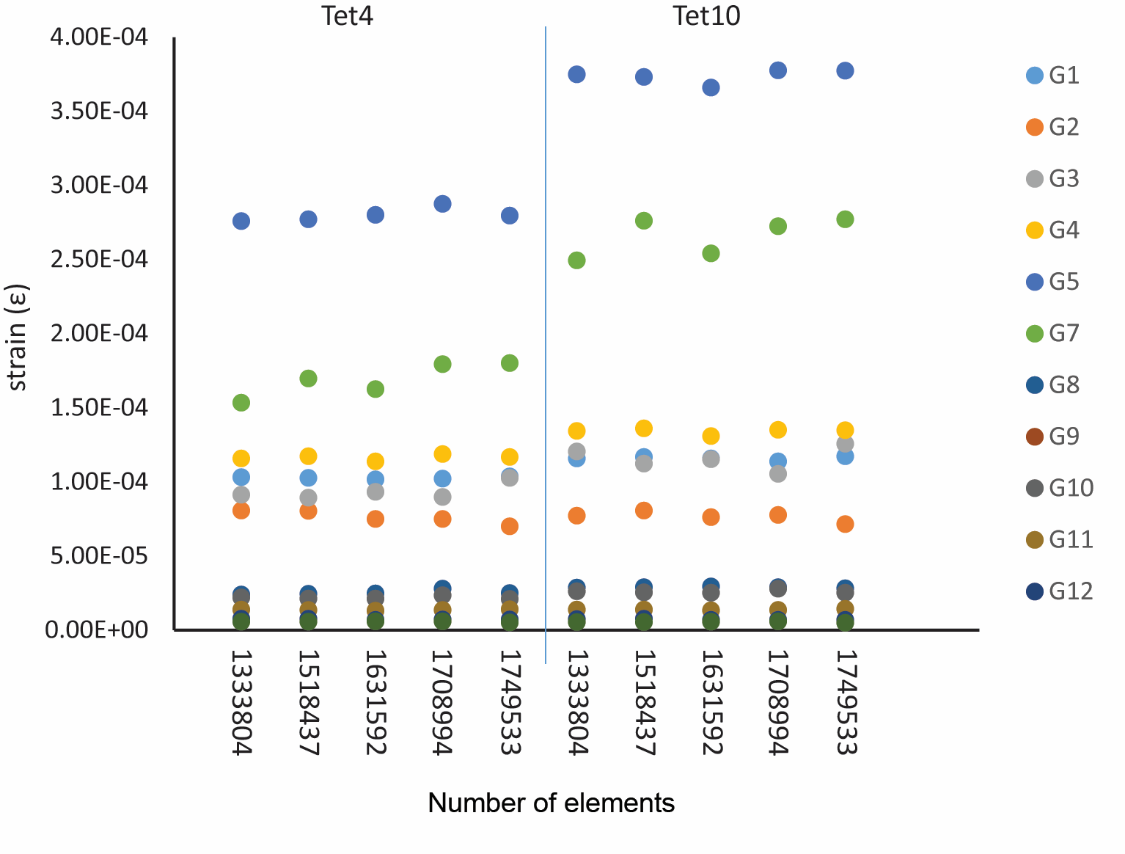


Table S2. E_min_ for the convergence testing of the ostrich model. All values reported in strain (ɛ).

|  | | Tet 4 | | | | | Tet10 | | | | |
| --- | --- | --- | --- | --- | --- | --- | --- | --- | --- | --- | --- |
| Number of elements | | 1333804 | 1518437 | 1631592 | 1708994 | 1749533 | 1333804 | 1518437 | 1631592 | 1708994 | 1749533 |
| Gauge number | 1 | -1.66E-04 | -1.72E-04 | -1.70E-04 | -1.72E-04 | -1.71E-04 | -1.85E-04 | -1.87E-04 | -1.85E-04 | -1.85E-04 | -1.84E-04 |
|  | 2 | -8.17E-05 | -8.18E-05 | -7.92E-05 | -8.37E-05 | -8.06E-05 | -8.76E-05 | -8.78E-05 | -8.57E-05 | -9.14E-05 | -8.76E-05 |
|  | 3 | -7.96E-05 | -7.75E-05 | -8.02E-05 | -7.80E-05 | -8.37E-05 | -9.23E-05 | -8.66E-05 | -8.94E-05 | -8.52E-05 | -9.28E-05 |
|  | 4 | -2.39E-04 | -2.44E-04 | -2.35E-04 | -2.45E-04 | -2.40E-04 | -2.32E-04 | -2.39E-04 | -2.31E-04 | -2.41E-04 | -2.38E-04 |
|  | 5 | -6.69E-04 | -6.74E-04 | -6.84E-04 | -7.08E-04 | -6.91E-04 | -9.61E-04 | -9.60E-04 | -9.61E-04 | -9.85E-04 | -9.73E-04 |
|  | 7 | -8.10E-05 | -8.50E-05 | -7.83E-05 | -8.71E-05 | -8.55E-05 | -1.07E-04 | -1.15E-04 | -1.07E-04 | -1.13E-04 | -1.16E-04 |
|  | 8 | -5.40E-05 | -5.74E-05 | -5.68E-05 | -6.43E-05 | -5.68E-05 | -7.10E-05 | -7.41E-05 | -7.41E-05 | -7.41E-05 | -7.06E-05 |
|  | 9 | -4.95E-05 | -4.77E-05 | -4.70E-05 | -5.59E-05 | -4.81E-05 | -6.21E-05 | -5.94E-05 | -5.75E-05 | -6.95E-05 | -5.94E-05 |
|  | 10 | -8.42E-06 | -9.07E-06 | -8.11E-06 | -7.52E-06 | -8.87E-06 | -8.17E-06 | -8.84E-06 | -7.83E-06 | -7.20E-06 | -8.63E-06 |
|  | 11 | -1.62E-05 | -1.58E-05 | -1.51E-05 | -1.62E-05 | -1.57E-05 | -1.56E-05 | -1.52E-05 | -1.48E-05 | -1.58E-05 | -1.53E-05 |
|  | 12 | -4.35E-06 | -4.32E-06 | -4.45E-06 | -4.85E-06 | -4.05E-06 | -4.38E-06 | -4.36E-06 | -4.45E-06 | -4.93E-06 | -4.16E-06 |
|  | 13 | -2.76E-06 | -2.81E-06 | -3.09E-06 | -3.20E-06 | -2.57E-06 | -2.71E-06 | -2.76E-06 | -3.05E-06 | -3.21E-06 | -2.49E-06 |

Figure S2. E_min_ for the convergence testing of the ostrich model. Each model is represented by a different column of coloured dots. Each colour is representative of a single gauge location. The line separates tet4 from tet10 models.


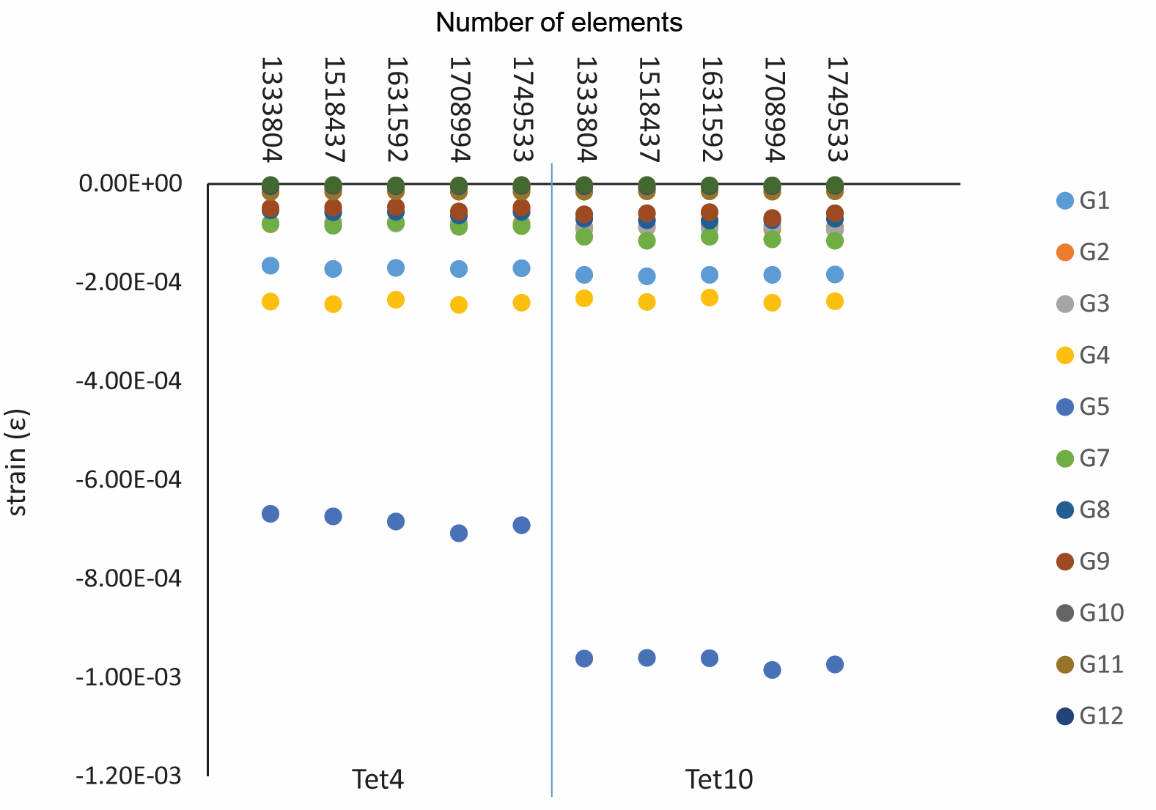


**Regression Analyses**

**Methods**

Data from the two experimental trials (trial 1 and trail 2) were regressed against each other for E_max_, E_min_, strain ratio and strain magnitudes. Strains at G5 (E_min_) and G7 (E_max_) were always exceptionally high. Because visual observation of the experiment confirmed that the skull undergoes extreme bending in the vicinity of these gauges (see also Gussekloo and Bout, 2005), we do not automatically believe that such high magnitudes are erroneous, but for the experiments where there is a such an outlier, the regressions were repeated with the outlier removed (G7 for E_max_, and strain ratio; G5 for E_min_) to see how these values affected the statistical results. R^2^ and p values were generated in PAST (Hammer et al. 2001).

In order to check whether the strain data from each group of material property trials (L1-L9, PH1-PH8, and N1-N3) could be averaged in the regression, the similarity of the means were tested using an ANOVA in PAST. The null hypothesis of similarity of means cannot be rejected for the pooled datasets L1-L9, PH1-PH8 in all metrics (E_max_, E_min_, strain ratios and orientations), and the means are the same for N1-N3 for all metrics except E_max_. The average FE results for each group of material property trials (L1-L9, PH1-PH8 and N1-N3) were therefore used to regress against the average experimental data, except when plotting E_max_, when each nanoindentation model (N1 to N3) was plotted separately.

**Results**

Trial 1 against 2

**E_max_**

Figure S3. Regression of *ex-vivo* maximum principal strains of Trial1 against Trial2.

Figure S4. Regression of *ex-vivo* maximum principal strains of Trial1 against Trial2 with G7 removed.

**E_min_**

Figure S5. Regression of *ex-vivo* minimum principal strains of Trial1 against Trial2.

Figure S6. Regression of *ex-vivo* minimum principal strains of Trial1 against Trial2 with G5 removed.

**Strain Ratios**

Figure S7. Regression of *ex-vivo* strain ratios of Trial1 against Trial2.

Figure S8. Regression of *ex-vivo* strain ratios of Trial1 against Trial2 with G7 removed.

**Strain orientations**

Figure S9. Regression of *ex-vivo* strain orientations of Trial1 against Trial2.

FE models against experimental data

**E_max_**

Figure S10. Regression of FE models against average experimental data for maximum principal strain (E_max_).

Literature: y = 0.0881x + 65.61, R² = 0.1077, p = 0.298.

Posthoc: y = 0.1771x + 122.23, R² = 0.1427, p = 0.226.

Nano mean: y = 0.2482x + 198.53, R² = 0.0766, p = 0.384.

Nano max: y = 0.1222x + 106.2, R² = 0.0694, p = 0.408.

Nano min: y = 0.6979x + 689.28, R² = 0.0462, p = 0.502.

Figure S11. Regression of FE models against average experimental data for maximum principal strain (E_max_) with G7 removed.

Literature: y = -1.2575x + 75.463, R² = 0.252, p = 0.140.

Posthoc: y = -2.1173x + 138.57, R² = 0.2439, p = 0.142.

Nano mean: y = -3.0999x + 228.1, R² = 0.1312, p = 0.293.

Nano max: y = -1.5487x + 121.12, R² = 0.1208, p = 0.316.

Nano min: y = -14.457x + 809.27, R² = 0.2108, p = 0.178.

**E_min_**

Figure S12. Regression of FE models against average experimental data for minimum principal strain (E_min_).

Literature: y = 0.5361x – 55.206, R² = 0.542, p = 0.00631.

Posthoc: y = 1.0767x – 89.492, R² = 0.6073, p = 0.00280.

Nano mean: y = 2.6946x – 322.07, R² = 0.4509, p = 0.0168.

Figure S13. Regression of FE models against average experimental data for minimum principal strain, G5 removed (E_min_).

Literature: y = 0.1252x – 81.128, R² = 0.0053, p = 0.831.

Posthoc: y = 0.1743x – 146.42, R² = 0.0035, p = 0.864.

Nano mean: y = 1.1148x – 322.07, R² = 0.0111, p = 0.758.

Figure S14. Regression of average experimental *ex-vivo* strain ratios against FE models.

Literature: y = 0.3359x + 0.9325, R² = 0.6333, p = 0.00197.

Posthoc: y = 0.3044x + 1.0319, R² = 0.5035, p = 0.00999.

Nano mean: y = 0.3308x + 0.9742, R² = 0.5498, p = 0.00572.

Figure S15. Regression of average experimental *ex-vivo* strain ratios against FE models with G7 removed.

Literature: y = 0.6755x + 0.9093, R² = 0.124, p = 0.302.

Posthoc: y = 0.728x + 1.003, R² = 0.1056, p = 0.344.

Nano mean: y = 1.1848x + 0.9159, R² = 0.2678, p = 0.107.

**Strain orientations**

Figure S16. Regression of average experimental *ex-vivo* strain orientations against FE models.

Literature: y = -0.0928x + 46.452, R² = 0.0464, p = 0.521.

Posthoc: y = -0.1072x + 47.126, R² = 0.0558, p = 0.460.

Nano mean: y = -0.1461x + 46.934, R² = 0.0646, p = 0.429.

Euclidean distances

Table S3. Euclidean distances from *ex-vivo* experimental measures to FE model data when possible outliers are removed. Data in bold are the closest to the experimental data for each specific metric. Asterisks (*) mark models that had previously been the best, and are no longer, when the outliers are removed.

|  | **Emax** | **Emin** | **Strain Ratio** |
| --- | --- | --- | --- |
| Literature |  |  |  |
| L1 | 358 | 410 | 3.61 |
| L2 | 415 | 582 | **2.59** |
| L3 | 350 | 459* | 2.69 |
| L4 | 391 | 567 | 3.61 |
| L5 | 337 | 457 | 3.67 |
| L6 | 328 | 453 | 3.64 |
| L7 | 381 | 548 | 3.63 |
| L8 | 377 | 543 | 3.60 |
| L9 | **274** | **423** | 3.16 |
|  |  |  |  |
| Posthoc |  |  |  |
| PH1 | 429 | 547 | 3.79 |
| PH2 | **420** | **546** | 4.13 |
| PH3 | 532 | 653 | 3.62 |
| PH4 | 528 | 640 | **3.51** |
| PH5 | 677 | 978 | 3.52 |
| PH6 | 676 | 1024 | 3.82 |
| PH7 | 679 | 1021 | 3.59* |
| PH8 | 874 | 1289 | 3.59 |
|  |  |  |  |
| Nanoindentation | |  |  |
| N1 | 1054 | 1725 | 3.52 |
| N2 | **566** | **868** | **3.40** |
| N3 | 3737 | 6494 | 3.51* |

**Reference**

Gussekloo, S.W.S., and Bout, R.G. 2005. Cranial kinesis in palaeognathous birds. *Journal of Experimental Biology* **208**, 3409-3419.

Hammer, Ø., Harper, D.A.T., Ryan, P.D. 2001. PAST: Paleontological statistics software package for education and data analysis. *Palaeontologia Electronica* 4(1) 1-9. http://palaeo-electronica.org/2001_1/past/issue1_01.htm
